# Transcatheter aortic valve replacement in heavily calcified aortic valve stenosis: a multicenter comparison

**DOI:** 10.1007/s00392-025-02611-w

**Published:** 2025-03-10

**Authors:** M. Saad, H. Seoudy, K. Bramlage, P. Bramlage, J. C. Voran, T. Puehler, G. Lutter, A. Allali, M. Landt, J. Frank, O. Bisht, M. Salem, H. Wienemann, M. Adam, T. Zeus, V. Veulemans, S. Bleiziffer, T. K. Rudolph, D. Frank

**Affiliations:** 1 Department of Internal Medicine III, Cardiology, Angiology and Intensive Care Medicine, University Hospital Schleswig-Holstein, Arnold-Heller-Str.3, Haus K3, 24105 Kiel, Germany; 2 DZHK (German Center for Cardiovascular Research), partner site Hamburg/Kiel/Lübeck, Kiel, Germany; 3 Institute for Pharmacology and Preventive Medicine, Cloppenburg, Germany; 4 Department of Cardiac and Vascular Surgery, University Clinical Center Schleswig-Holstein (UKSH), Kiel, Germany; 5 Heart Center Segeberger Kliniken GmbH, Am Kurpark 1, 23795 Bad Segeberg, Germany; 6 Klinik III für Innere Medizin-Kardiologie, Herzzentrum der Universität Köln, Kerpener Str. 62, 50937 Köln, Germany; 7 Klinik für Kardiologie, Pneumologie & Angiologie, Universitätsklinikum Düsseldorf, 40225 Düsseldorf, Germany; 8 Clinic for General and Interventional Cardiology/Angiology, Herz- und Diabeteszentrum NRW, 32545 Bad Oeynhausen, Germany

**Keywords:** Transcatheter aortic valve replacement, Aortic valve stenosis, Paravalvular leak, Calcification

## Abstract

**Background:**

Heavy calcifications in severe aortic stenosis (AS) pose a major challenge in patients undergoing transcatheter aortic valve replacement (TAVR). Only a few studies have addressed the performance of different transcatheter heart valves (THV) in this subgroup of patients.

**Objectives:**

We aimed to investigate the outcomes of the self-expanding Medtronic CoreValve Evolut valve frame and the balloon-expandable Edwards SAPIEN-3/3 Ultra THV in this challenging patient population.

**Materials and methods:**

This was a multicenter registry including a total of 1513 patients with heavily calcified AS undergoing TAVR. The primary endpoint was the incidence and degree of paravalvular leak (PVL) after TAVR. Secondary endpoints were post-implant hemodynamics as well as clinical endpoints according to the VARC-3 definitions.

**Results:**

The CoreValve Evolut R but not the Evolut PRO showed significantly higher rates of PVL compared to the SAPIEN-3/3 Ultra (44.8% vs. 29.5% for mild PVL, p < 0.001), while there was no significant difference in ≥ moderate PVL between both groups (p = 0.399). The CoreValve Evolut R and Evolut PRO showed superior THV hemodynamics compared to the SAPIEN-3/3 Ultra group. These findings were confirmed in a propensity score-matched analysis. There were no significant differences regarding short-term outcomes including permanent pacemaker implantation and all-cause mortality between the three groups.

**Conclusion:**

In patients with severely calcified AS, both CoreValve Evolut PRO and SAPIEN-3/3 Ultra THV showed lower rates of PVL than the CoreValve Evolut R. The self-expanding CoreValve platform had superior post-implant hemodynamics than the SAPIEN-3/3 Ultra system.

**Graphical Abstract:**

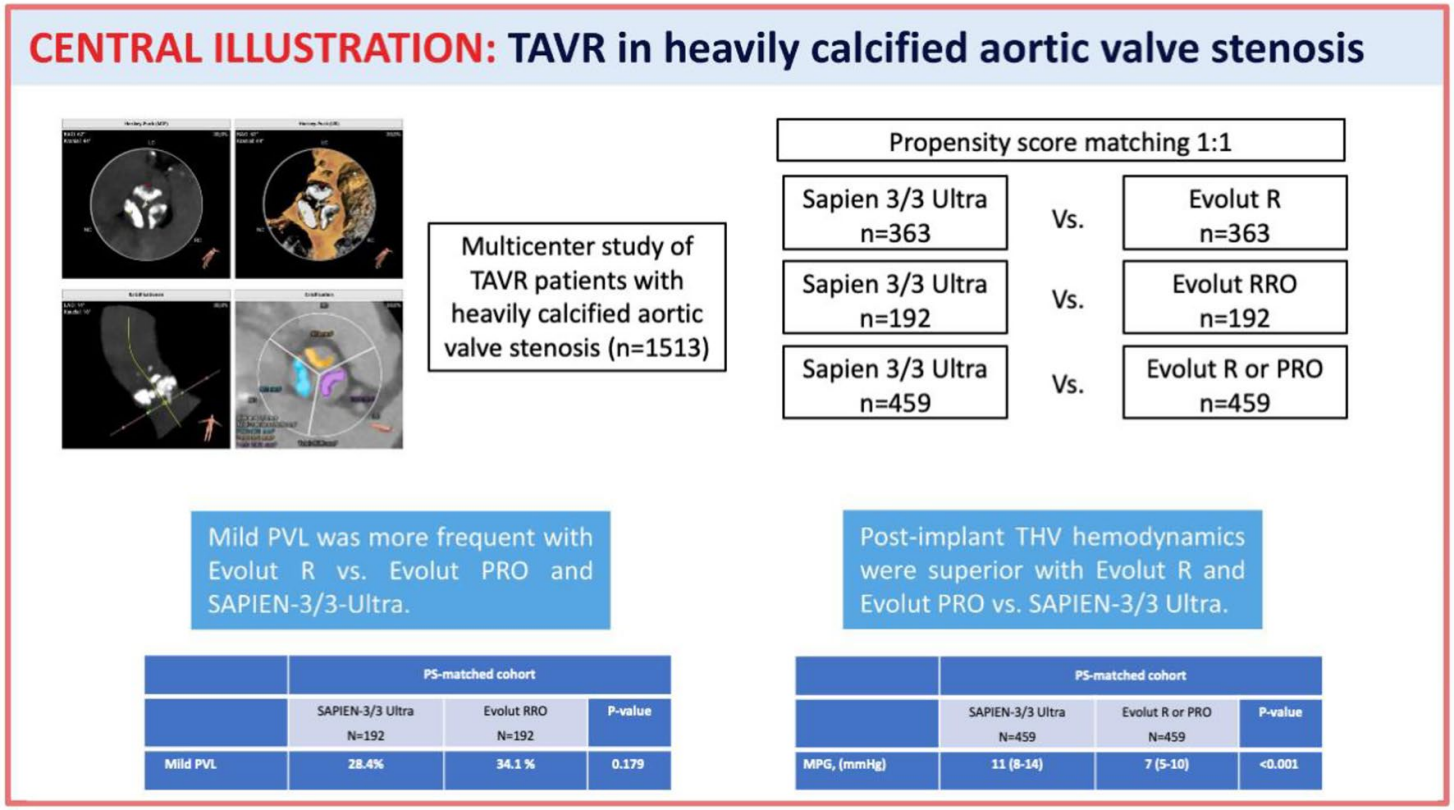

## Introduction

Transcatheter aortic valve replacement (TAVR) has emerged as an alternative to surgical aortic valve replacement (SAVR) for the majority of patients with severe symptomatic aortic valve stenosis (AS) across the whole spectrum of surgical risk [1, 2]. While various transcatheter heart valve (THV) devices are available on the market, the CoreValve Evolut^®^ (Medtronic, Minneapolis, Minnesota) and the Edwards SAPIEN^™^ (Edwards Lifescience, Irvine, USA) systems are the most commonly used THVs worldwide [3].

TAVR can be safely performed in an expanding population with aortic valve disease. However, the presence of heavy calcifications in patients with AS remains to pose a major challenge in clinical practice. In particular, heavy calcifications are a well-recognized risk factor for paravalvular leak (PVL) after TAVR by potentially impairing complete apposition and sealing of the THV [4]. This has important prognostic implications, as several studies have confirmed more than moderate PVL to be a strong predictor of both morbidity and mortality after TAVR [5–8]. Substantial technological advancements in THV designs have led to a significant reduction of PVL and higher device success rates using new-generation devices [9–11]. The Evolut R includes a skirt extending at the in-flow aspect of the valve. The newer Evolut PRO/PRO + design adds a porcine pericardial tissue wrap around the outer sealing zone of its frame. Similarly, the SAPIEN-3/3 Ultra THV includes an outer skirt sealing the gap between the native annulus and the THV [3].

Despite the widespread adoption of TAVR, data reporting a head-to-head comparison of the new generation THV in severely calcified AS are still scarce. Thus, the aim of the present study was to compare the CoreValve Evolut and SAPIEN-3 platform with a focus on the incidence of PVL after TAVR in a real-world, industry-independent registry.

## Methods

### Study design and patient population

**Fig. 1:**
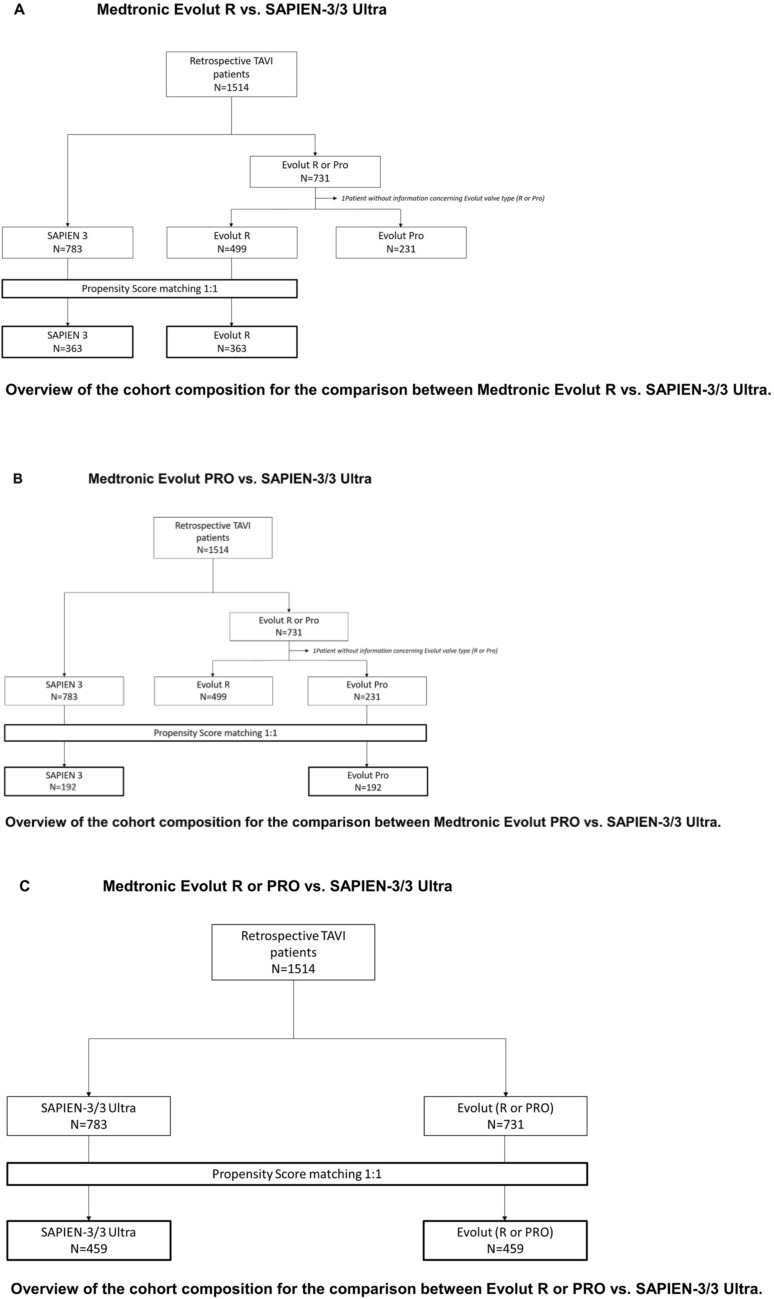
**A** Medtronic Evolut R vs. SAPIEN-3/3 Ultra. ** B** Medtronic Evolut PRO vs. SAPIEN-3/3 Ultra. ** C** Medtronic Evolut R or PRO vs. SAPIEN-3/3 Ultra

This was a multicenter, retrospective, non-randomized cohort study including patient data from 5 high-volume TAVR centers in Germany. A database search was performed for all patients with symptomatic, heavily calcified AS who underwent TAVR between July 2013 and January 2021. A total of 1513 patients were eligible for final analysis. Based on the THV type, patients were further categorized into three groups: (1) Evolut R, (2) Evolut PRO and (3) SAPIEN-3/3 Ultra. To adjust for baseline differences between the groups, propensity score-matched analyses were applied (Fig. 1 A/B/C).

All patients received a high-resolution multidetector computed tomography (MDCT) scan prior to the procedure. Heavily calcified AS was defined as a calcium score of > 750 mm^3^ using 3mensio software (Pie Medical Imaging, Bilthoven, the Netherlands) or an Agatston Score > 3000 AU [2, 12]. Further inclusion criteria were the use of the Evolut R/PRO or SAPIEN-3/3 Ultra THV and written informed consent. Exclusion criteria comprised valve-in-valve procedures, the use of THVs other than the above-mentioned devices and missing data. The primary endpoint was the incidence and degree of PVL after TAVR. Secondary endpoints were peak and mean pressure gradients across the THV, THV effective orifice area as well as clinical endpoints according to the VARC-3 definitions [13]. Follow-up after discharge was obtained during routine visits to the cardiology outpatient clinic or by telephone calls. The study was conducted in accordance with the Declaration of Helsinki and was approved by the local ethics boards. All patients provided informed consent to the procedure before intervention.

### Procedural details

All TAVR procedures were performed by experienced and certified operators according to the standards of best clinical practice using either the CoreValve Evolut R/PRO or SAPIEN-3/3 Ultra THV. The choice of THV type was left at the discretion of the implanting operator after a comprehensive preprocedural workup. Accordingly, the necessity of pre-dilatation and post-dilatation were also determined by the implanting operator. To allow for good expansion of the SEV and to avoid post dilatation, predilatation was done when severe calcification was observed especially with non-homogenous distribuation of the leaflet calcification. During the TAVR procedure, unfractionated heparin was administered to achieve an activated clotting time of 250–300 s. Closure of the vascular access was usually performed using two Perclose ProGlide™ vascular closure systems (Abbott Laboratories, Chicago, IL, USA) or the MANTA^®^ vascular closure device (Teleflex, Morrisville, NC, USA).

### Statistical analyses

Standard descriptive statistics were used. Categorical variables were presented as absolute values and frequencies (%). A Kolmogorov–Smirnov-test was used to test continuous variables for normal distribution. Accordingly, continuous variables were shown as means with standard deviations (SDs) or median and interquartile range (IQR). All descriptive statistics were based on available cases. Intergroup comparisons were done using t-test, Mann–Whitney U-test for continuous variables, and Fisher’s exact or Chi-square test for categorical variables, as appropriate. All tests were two-sided and a p-value < 0.05 was considered statistically significant. The statistical analysis was performed using SPSS Version 29.0 (Armonk, NY, IBM Corp., USA).

The study included three propensity score-matched analyses, as follows:

A Medtronic Evolut R vs. SAPIEN-3/3 Ultra (Fig. 1)

The propensity score for each patient was calculated by logistic regression with adjustment for 10 key baseline variables including age, gender, body mass index, logistic EuroSCORE, hypertension, atrial fibrillation, dyslipidemia, NYHA class III or IV on admission, aortic valve area, and aortic valve calcium score. A difference in propensity score of 1% (0.01) was tolerated when matching patients 1:1.

B Medtronic Evolut PRO vs. SAPIEN-3/3 Ultra (Fig. 1)

The propensity score for each patient was calculated by logistic regression with adjustment for 12 key baseline variables including age, gender, body mass index, logistic EuroSCORE, hypertension, atrial fibrillation, dyslipidemia, diabetes, peripheral artery disease, NYHA class III or IV on admission, aortic valve area, and aortic valve calcium score. A difference in propensity score of 1.5% (0.015) was tolerated when matching patients 1:1.

C Medtronic Evolut PRO or R vs. SAPIEN-3/3 Ultra (Fig. 1)

The propensity score for each patient was calculated by logistic regression with adjustment for 10 key baseline variables, including age, gender, body mass index, logistic EuroSCORE, hypertension, atrial fibrillation, dyslipidemia, peripheral artery disease, aortic valve area, and aortic valve calcium score. A difference in propensity score of 3.0% (0.03) was tolerated when matching patients 1:1.

## Results

The present study included a total of 1513 patients with heavily calcified AS undergoing TAVR. The CoreValve Evolut R was implanted in 499 patients (33.0%), the Evolut PRO in 231 patients (15.3%) and the SAPIEN-3/3 Ultra THV in 783 patients (51.7%).

### Patient characteristics

Baseline characteristics of the total unmatched cohort are summarized in Table 1. The mean age in the SAPIEN-3/3 Ultra group was slightly lower than in the two CoreValve groups (81 vs. 82 years, p = 0.012). In addition, the SAPIEN-3/3 Ultra group included more male patients and showed higher rates of atrial fibrillation and a higher logistic EuroSCORE, while the prevalence of dyslipidemia was significantly lower than in the CoreValve groups. Left ventricular outflow tract calcifications were more frequently noted in the CoreValve Evolut PRO and SAPIEN-3/3 Ultra group compared to the Evolut R group. With regards to procedural factors, procedural time and contrast volume were significantly higher and pre- and postdilatation more frequently used in the two CoreValve groups compared to the SAPIEN-3/3 Ultra group. After propensity score matching, there were no significant differences in the baseline characteristics between the three different THV groups. Regarding procedural characteristics predilatation, postdilatation and procedural time, but not contrast volume were still higher in both CoreValve groups compared to the SAPIEN-3/3 Ultra group.

**Table 1:** Baseline characteristics

Variable	SAPIEN-3/3 ultra (n = 783)	Evolut R (n = 499)	Evolut PRO (n = 231)	*P*-value
Age, (years)	81 ± 6	82 ± 5	82 ± 5	0.012
Male, n (%)	563/761 (74%)	280/486 (58%)	109/218 (50%)	< 0.001
Hypertension, n (%)	680/748 (91%)	413/469 (88%)	180/208 (87%)	0.256
Diabetes mellitus, n (%)	202/748 (27%)	118/469 (25%)	69/209 (33%)	0.103
Dyslipidemia, n (%)	492/749 (66%)	365/469 (78%)	166/209 (79%)	< 0.001
Body mass index, (kg/m^2^)	27 ± 5	26 ± 5	27 ± 5	0.015
Ischemic heart disease, n (%)	467/749 (62%)	285/469 (61%)	122/209 (58%)	0.561
Atrial fibrillation, n (%)	294/719 (41%)	166/457 (36%)	60/199 (30%)	0.016
Peripheral artery disease, n (%)	109/749 (15%)	58/468 (12%)	20/209 (10%)	0.144
COPD, n (%)	135/747 (18%)	90/469 (19%)	38/209 (18%)	0.882
History of stroke, n (%)	52/592 (9%)	33/435 (8%)	13/205 (6%)	0.505
Permanent pacemaker, n (%)	89/749 (12%)	50/468 (11%)	18/209 (9%)	0.395
STS-score, (%)	5 ± 5	5 ± 4	5 ± 3	0.741
Logistic EuroSCORE, (%)	20 ± 15	19 ± 13	16 ± 12	< 0.001
EuroSCORE II, (%)	6 ± 7	5 ± 6	5 ± 4	0.040
NYHA Class I, n (%) II, n (%) III, n (%) IV, n (%)	33/747 (4%) 239/747 (32%) 420/747 (56%) 55/747 (8%)	16/468 (3%) 129/468 (28%) 288/468 (62%) 35/468 (7%)	9/209 (4%) 78/209 (38%) 113/209 (54%) 9/209 (4%)	0.229
hsTNT—before TAVR, (pg/ml)	125 ± 762	64 ± 204	142 ± 513	0.248
NT-proBNP—before TAVR, (pg/ml)	4708 ± 7235	5447 ± 17479	6828 ± 14282	0.354
eGFR (ml/min/1.73 cm^2^)	58 ± 22	56 ± 20	56 ± 20	0.315
MPG—invasive, (mmHg)	45 ± 18	42 ± 17	46 ± 38	0.062
LVEF—echo before TAVR, (%)	52 ± 12	53 ± 11	53 ± 9	0.246
AVA—echo before TAVR, (cm^2^)	0.7 ± 0.2	0.6 ± 0.3	0.6 ± 0.3	0.010
LVOT calcification, n (%)	349/498 (70%)	265/429 (62%)	154/205 (75%)	< 0.001
Bicuspid aortic valve, n (%)	24/581 (4%)	23/435 (5%)	7/205 (3%)	0.502
Vascular access Transfemoral, n (%) Transaortic, n (%) Transapical, n (%) Subclavian, n (%)	727/782 (93%) 21/782 (3%) 27/782 (3%) 7/782 (1%)	475/499 (95%) 14/499 (3%) – 10/499 (2%)	219/231 (95%) 10/231 (4%) – 2/231 (1%)	
Predilatation, n (%)	247/765 (32%)	237/490 (48%)	106/220 (48%)	< 0.001
Postdilatation, n (%)	78/769 (10%)	196/490 (40%)	101/223 (45%)	< 0.001
Contrast volume, (ml)	105 ± 36	115 ± 46	117 ± 45	< 0.001
Procedural time, (minutes)	66 ± 29	74 ± 29	68 ± 26	< 0.001

Continuous data are presented as mean ± SD

AVA: aortic valve area; COPD: chronic obstructive pulmonary disease; GFR: glomerular filtration rate; hsTNT: high-sensitive Troponin T; LVEF: left ventricular ejection fraction; LVOT: left ventricular outflow tract; MPG: mean pressure gradient; NT-proBNP: N-terminal pro B-type Natriuretic Peptide; NYHA: New York heart association; TAVR: transcatheter aortic valve replacement

### PVL and THV hemodynamics

Data on PVL and THV hemodynamics are presented in Table 2, 3 and 4. The SAPIEN-3/3 Ultra showed significantly lower rates of PVL compared to the CoreValve Evolut R group (p < 0.001). This was driven by a significantly higher incidence of mild PVL in the CoreValve Evolut R group (44.8% for Evolut R vs. 29.5% for SAPIEN-3/3 Ultra), while there was no significant difference in moderate or severe PVL between both groups (p = 0.399). Comparing the SAPIEN3/3 Ultra with the CoreValve Evolut PRO, there was no significant difference both in total PVL (41.2 vs. 49.5% respectively, p = 0.084) and in moderate or severe PVL (11.7 vs. 15.1% respectively, p = 0.213).

**Table 2:** Paravalvular leak and valve hemodynamics—SAPIEN-3/3 Ultra vs. CoreValve Evolut R

	Unmatched cohort			PS-matched cohort		
	SAPIEN-3/3 ultra N = 783	Evolut R N = 499	p-value	SAPIEN-3/3 ultra N = 363	Evolut R N = 363	P-value
	n/N (%) or mean ± SD/median (IQR)	n/N (%) or mean ± SD/median (IQR)		n/N (%) or mean ± SD/median (IQR)	n/N (%) or mean ± SD/median (IQR)	
PVL	566	339	< 0.001	263	297	< 0.001
None, n (%)	333 (58.8)	141 (41.6)		149 (56.7)	123 (41.4)	
Mild, n (%)	167 (29.5)	152 (44.8)		75 (28.5)	132 (44.4)	
Moderate, n (%)	65 (11.5)	42 (12.4)		38 (14.4)	40 (13.5)	
Severe, n (%)	1 (0.2)	4 (1.2)		1 (0.4)	2 (0.7)	
PVL moderate/severe, n (%)	66 (11.7)	46 (13.6)	0.399	39/263 (14.8)	42/297 (14.1)	0.817
PPG, (mmHg)	21 ± 9 21 (15–27) 511	15 ± 7 13 (9–18) 311	< 0.001	22 ± 9 22 (16–27) 234	15 ± 7 13 (9–18) 266	< 0.001
MPG, (mmHg)	12 ± 5 11 (8–14) 589	8 ± 4 7 (5–10) 350	< 0.001	12 ± 5 11 (9–14) 277	8 ± 4 7 (5–10) 292	< 0.001
THV area, (cm^2^)	1.7 ± 0.4 1.60 (1.40–1.90) 446	2 ± 0.6 1.90 (1.60–2.25) 193	< 0.001	1.67 ± 0.42 1.60 (1.40–1.80) 186	2.00 ± 0.57 1.90 (1.60–2.30) 166	< 0.001

MPG: mean pressure gradient; PPG: peak pressure gradient; PVL: paravalvular leak; THV-EOA: transcatheter heart valve effective orifice area

**Table 3:** Paravalvular leak and valve hemodynamics—SAPIEN-3/3 Ultra vs. Evolut PRO

	Unmatched cohort			PS-matched cohort		
	SAPIEN-3/3 ultra N = 783	Evolut PRO N = 231	p-value	SAPIEN-3/3 ultra N = 192	Evolut PRO N = 192	p-value
	n/N (%) or mean ± SD/median (IQR)	n/N (%) or mean ± SD/median (IQR)		n/N (%) or mean ± SD/median (IQR)	n/N (%) or mean ± SD/median (IQR)	
PVL	566	192	0.084	134	179	0.179
None, n (%)	333 (58.8)	97 (50.5)		82 (61.2)	90 (50.3)	
Mild, n (%)	167 (29.5)	66 (34.4)		38 (28.4)	61 (34.1)	
Moderate, n (%)	65 (11.5)	27 (14.1)		14 (10.4)	26 (14.5)	
Severe, n (%)	1 (0.2)	2 (1.0)		0 (0)	2 (1.1)	
PVL mod/severe, n (%)	66 (11.7)	29 (15.1)	0.213	14 (10.4)	28 (15.6)	0.182
PPG, (mmHg)	21 ± 9 21 (15–27) 511	16 ± 8 14 (10–21) 156	< 0.001	22 ± 10 20 (15–27) 119	15 ± 8 14 (10–21) 144	< 0.001
MPG, (mmHg)	12 ± 5 11 (8–14) 589	9 ± 4 8 (5–11) 182	< 0.001	12 ± 5 11 (8–14) 142	9 ± 4 8 (5–11) 168	< 0.001
THV area, (cm^2^)	1.7 ± 0.4 1.60 (1.40–1.90) 446	1.9 ± 0.5 1.80 (1.50–2.15) 137	< 0.001	1.69 ± 0.44 1.60 (1.40–1.89) 96	1.87 ± 0.48 1.80 (1.50–2.18) 128	0.002

MPG: mean pressure gradient; PPG: peak pressure gradient; PVL: paravalvular leak; THV-EOA: transcatheter heart valve effective orifice area

**Table 4:** Paravalvular leak and valve hemodynamics—SAPIEN-3/3 Ultra vs. Evolut R or PRO

	Unmatched cohort			PS-matched cohort		
	SAPIEN-3/3 ultra N = 783	Evolut R or PRO N = 731	p-value	SAPIEN-3/3 ultra N = 459	Evolut R or PRO N = 459	p-value
	n/N (%) or mean ± SD/median (IQR)	n/N (%) or mean ± SD/median (IQR)		n/N (%) or mean ± SD/median (IQR)	n/N (%) or mean ± SD/median (IQR)	
PVL	566	532	< 0.001	331	421	< 0.001
None, n (%)	333 (58.8)	239 (44.9)		193 (58.3)	155 (36.8)	
Mild, n (%)	167 (29.5)	218 (41.0)		98 (29.6)	196 (46.6)	
Moderate, n (%)	65 (11.5)	69 (13.0)		40 (12.1)	66 (15.7)	
Severe, n (%)	1 (0.2)	6 (1.1)		0 (0)	4 (1.0)	
PVL mod/severe, n (%)	66 (11.7)	75 (14.1)	0.228	40 (12.1)	70 (16.6)	0.080
PPG, (mmHg)	22 ± 9 21 (15–27) 511	15 ± 7 14 (9–20) 468	< 0.001	21.8 ± 8.9 21 (15;27) 291	14.8 ± 7.3 14 (9.3;19.2) 371	< 0.001
MPG, (mmHg)	12 ± 5 11 (8–14) 589	8 ± 4 7 (5–10) 533	< 0.001	11.8 ± 4.9 11 (8;14) 343	8 ± 4 7 (5–10) 404	< 0.001
THV area, (cm^2^)	1.71 ± 0.44 1.60 (1.40–1.90) 446	1.93 ± 0.53 1.84 (1.60;2.20) 331	< 0.001	1.70 ± 0.43 1.60 (1.40;1.90) 242	1.95 ± 0.55 1.90 (1.60;2.20) 257	< 0.001

MPG: mean pressure gradient; PPG: peak pressure gradient; PVL: paravalvular leak; THV-EOA: transcatheter heart valve effective orifice area

The CoreValve Evolut R and Evolut PRO showed superior THV hemodynamics compared to the SAPIEN-3/3 Ultra with regards to peak pressure gradient (15 ± 7 mmHg for Evolut R vs. 16 ± 8 mmHg for Evolut PRO vs. 21 ± 9 mmHg for SAPIEN-3/3 Ultra, p < 0.001), mean pressure gradient (8 ± 4 mmHg for Evolut R vs. 9 ± 4 mmHg for Evolut PRO vs. 12 ± 5 mmHg for SAPIEN-3/3 Ultra, p < 0.001) and effective orifice area (2 ± 0.6 cm^2^ for Evolut R vs. 1.9 ± 0.5 cm^2^ for Evolut PRO vs. 1.7 ± 0.4 cm^2^ for SAPIEN-3/3 Ultra, p < 0.001). These findings were confirmed in the propensity score-matched analysis.

### Clinical outcomes

Clinical outcomes are shown in Table 5. There was no significant difference between the three groups regarding all-cause mortality, cardiovascular mortality, new permanent pacemaker implantation, stroke with disability, myocardial infarction, major bleeding, major access site complications and conversion to open surgery at 30 days. The SAPIEN-3/3 Ultra group did, however, show significantly higher rates of pericardial tamponade when compared to the CoreValve Evolut R (1.4 vs 0%, respectively, p = 0.024) and significantly higher rates of acute kidney injury when compared to the CoreValve Evolut PRO (8.3 vs 3.9%, respectively, p = 0.021).

**Table 5:** 30 day follow-up

	SAPIEN-3/3 ultra (n = 783)	Evolut R (n = 499)	Evolut PRO (n = 231)	S3vsR p-value	S3vsPRO p-value
All-cause mortality, n (%)	19/749 (2.5%)	6/469 (1.3%)	7/209 (3.3%)	0.150	0.478
Cardiovascular mortality, n (%)	13/19 (68.4%)	6/6 (85.7%)	4/7 (57.1%)	0.629	0.661
Myocardial infarction, n (%)	1/592 (0.2%)	1/435 (0.2%)	0/205 (0%)	1.000	1.000
Stroke, n (%) Stroke with disability, n (%)	13/749 (1.7%) 9/11 (81.8%)	8/469 (1.7%) 3/8 (37.5%)	10/209 (4.8%) 4/9 (44.4%)	1.000 0.074	0.019 0.160
Bleeding, n (%) ≥ type2, n (%) Type 1, n (%)	75/749 (10%) 54/75 (72%) 21/75 (28%)	62/469 (13.2%) 37/62 (60%) 25/62 (40%)	19/209 (9.1%) 16/19 (84.2%) 3/19 (15.8%)	0.093 0.148 0.148	0.793 0.382 0.382
Acute kidney injury, n (%)	65/783 (8.3%)	31/499 (6.2%)	9/231 (3.9%)	0.192	0.021
Access site complications, n (%) Major complications, n (%) Minor complications, n (%)	55/749 (7.3%) 24/55 (43.6%) 31/55 (56.4%)	63/469 (13.4%) 23/63 (36.5%) 40/63 (63.5%)	19/209 (9.1%) 7/19 (36.8%) 12/19 (63.2%)	0.001 0.456 0.456	0.383 0.788 0.788
New PPI, n (%)	100/660 (15.2%)	77/449(17.1%)	31/213 (14.6%)	0.373	0.832
Conversion to open surgery, n (%)	3/749 (0.4%)	0/469 (0%)	1/209 (0.5%)	0.289	1.000
Pericardial tamponade	8/592 (1.4%)	0/435 (0%)	0/205 (0%)	0.024	0.122
Bailout valve-in-valve	6/482 (1.2%)	1/428 (0.2%)	1/205 (0.5%)	0.128	0.681

PPI: permanent pacemaker implantation

## Discussion

This was a real-world, industry-independent, multicenter study including 1513 patients with severely calcified AS undergoing TAVR using either the CoreValve Evolut R, Evolut PRO or the Edwards SAPIEN-3/3 Ultra THV. The main findings of the study are: (1) compared to the SAPIEN-3/3 Ultra, mild PVL was more frequently found in the CoreValve Evolut R, but not Evolut PRO; (2) both Medtronic Evolut R and Evolut PRO showed superior post-implant hemodynamics than the SAPIEN-3/3 Ultra; (3) there were no significant differences regarding short-term mortality between the three groups.

### Incidence and prognostic impact of PVL after TAVR

Based on previous studies, there is general consensus that PVL after TAVR is significantly associated with the severity of aortic valve calcification [14–18]. This may be explained with the inability of the THV to be fully and symmetrically expanded in the presence of heavy calcifications which might impede tight sealing. In a large meta-analysis evaluating PVL post TAVR using first-generation THVs, self-expanding prostheses showed higher rates of moderate to severe PVL compared to balloon-expandable THVs [19]. Due to technological advances, the rates of PVL have substantially decreased using newer generation THVs compared to first-generation THVs. In a study comparing the incidence of PVL between patients who underwent TAVR with first-generation and new generation prostheses, lower rates of PVL were found in the new generation group compared to the first-generation group [20]. In this study, patients treated with the Medtronic Evolut R had rates of 8.6% for moderate and 2.5 for severe PVL, while patients receiving a SAPIEN-3 had a rate of 6.7% for moderate PVL and no severe PVL. Similarly, another study reported that greater than mild PVL occurs less frequently in patients treated with newer generation THVs with a rate of 5.3% for the CoreValve Evolut R and none for the SAPIEN-3 [21]. In our study, higher incidences of PVL were observed both for the CoreValve Evolut R (12.4% for moderate and 1.2% for severe PVL) and for the SAPIEN-3/3-Ultra (11.5% for moderate and 0.2% for severe PVL). These higher incidences of PVL in our cohort can be explained with the severe aortic valve calcifications in our patient population.

Notably, compared with the CoreValve Evolut R, the CoreValve Evolut PRO showed significantly lower rates of PVL. The CoreValve Evolut PRO is the third generation self-expanding THV which is built on the CoreValve Evolut R valve platform, but being modified by the addition of an external pericardial tissue wrap around the valve inflow, designed to minimize PVL. In the initial study of the CoreValve Evolut PRO performed in 60 patients in the United States, 72.4% of patients had no or trace PVL and 27.6% had mild PVL [22]. No moderate or severe PVL were noted. Similarly, in a report from the STS/ACC TVT registry comparing the CoreValve Evolut R and CoreValve Evolut PRO in a propensity score-matched analysis, the rate of moderate or severe PVL was 5.4% in the CoreValve Evolut R group and 3.4% in the CoreValve Evolut PRO [23]. Our study is in line with these previously published data demonstrating a higher rate of PVL for the CoreValve Evolut R compared to the CoreValve Evolut PRO and SAPIEN-3/3 Ultra [24–26]. Total rate of PVL across all THV types was significantly more frequent in our study which, again, is explained by the severely aortic valve calcifications in our patient cohort.

### Short-term clinical outcomes

In our study, SAPIEN-3/3 Ultra showed significantly higher rates of pericardial tamponade when compared to the CoreValve Evolut R (1.4 vs 0%, respectively, p = 0.024). This may be related to the use of balloon-expandable THV in severely calcified annuli in our cohort. Moreover, the SAPIEN-3/3 Ultra group showed significantly higher rates of acute kidney injury when compared to the CoreValve Evolut PRO (8.3 vs 3.9%, respectively, p = 0.021). There were no significant differences in other clinically relevant short-term outcomes. In particular, the incidence of new permanent pacemaker was comparable between the three groups (15.2% for SAPIEN-3/3 Ultra, 17.1% for CoreValve Evolut R and 14.6% for CoreValve Evolut PRO, p = 0.832). Our findings are mainly consistent with two previous studies demonstrating similar clinical outcomes between the CoreValve Evolut R and the SAPIEN-3 [26, 27]. Interestingly, however, the total rate of new permanent pacemaker implantation in our patient cohort was lower than in the previously mentioned studies. In contrast, the CENTER-collaboration registry comparing the CoreValve Evolut R to the SAPIEN-3 demonstrated a higher rate of conversion to open heart surgery, stroke, and new permanent pacemaker implantation in the CoreValve Evolut R group, while major or life-threatening bleeding were more frequent in the SAPIEN-3 group [28]. Mortality did not differ between the two groups.

### THV hemodynamics

In our analysis, both CoreValve Evolut R and CoreValve Evolut PRO groups showed superior post-implant hemodynamics compared to the SAPIEN-3/3 Ultra with regard to peak and mean pressure gradients as well as effective orifice area. This difference in the hemodynamics is mainly related to the supra-annular design of the CoreValve system, which had previously been demonstrated to yield superior hemodynamics compared to balloon-expanding valve systems such as the SAPIEN-3/3 Ultra platform [29, 30]. Consistent with our results, the OCEAN-TAVI registry reported superior hemodynamics of the CoreValve Evolut R compared to the SAPIEN-3 in patients with a small aortic annulus [31]. Another study demonstrated that the CoreValve Evolut R achieved larger effective orifice area and lower mean pressure gradients than the SAPIEN-3 [30]. Similar results were reported in the CHOICE-Extend registry where the mean pressure gradient was 7 ± 3 mm Hg for the CoreValve Evolut R compared to 12 ± 4 mm Hg for SAPIEN-3 [27]. Interestingly, the addition of the outer pericardial wrap did not show a negative impact on the hemodynamic performance of the CoreValve Evolut PRO when compared to the SAPIEN-3, even in the high-risk population of patients with severely calcified AS. This is consistent with the findings from the initial CoreValve Evolut PRO Study and data reported from the STS/ACC TVT registry in the general TAVR population [22, 23].

Whether the inferior hemodynamics of the SAPIEN-3/3 Ultra may translate into worse long-term outcomes such as earlier valve deterioration in patients with heavily calcified AS is beyond the scope of our study and thus warrants further investigation. A recent report by Herrmann et al. demonstrated that TAVR patients with severe, but not moderate patient-prosthesis mismatch are affected by higher rates of heart failure rehospitalization and increased late mortality compared to patients without patient-prosthesis mismatch [32]. Further, long-term, head-to-head comparison using standardized criteria between supra-annular and intra-annular valves are needed.

## Conclusion

In patients with severely calcified AS, both CoreValve Evolut PRO and SAPIEN-3/3 Ultra THV showed lower rates of PVL than the CoreValve Evolut R. The self-expanding CoreValve platform had superior post-implant hemodynamics than the SAPIEN-3/3 Ultra system.

## Study limitations

This is a multi-center observational study which is limited by its retrospective, non-randomized design. The decision whether to use the CoreValve THV or the SAPIEN-3/3 Ultra THV was at the discretion of the implanting TAVR operator. The selection of the prosthesis type is influenced by the anatomy of the aortic valve, and severity and distribution of calcification, especially in LVOT, which may have led to bias in choosing the type of THV. One of the major limitations of our study is the use of contrast and non-contrast enhanced methods of calcium measurement in the different centers leading to a high likelihood of under- or overestimation. While immediate post-implant hemodynamics were superior for the CoreValve platform, it remains debatable if this translates into clinically significant differences in patient outcomes, such as heart failure symptoms, cardiac-related rehospitalization rates, quality of life and long-term THV durability. Thus, both measured and unmeasured confounding factors may limit the conclusions that can be drawn from this analysis. Despite these limitations, this study provides important data on the use of TAVR in patients with heavily calcified AS.

## Data Availability

The authors confirm that the data supporting the findings of this study are available from the corresponding author [MS] on request.

## Abbreviations

AS: Aortic valve stenosis
AVC: Aortic valve calcification
PPI: Permanent pacemaker implantation
PVL: Paravalvular leak
TAVR: Transcatheter aortic valve replacement
THV: Transcatheter heart valves
VARC: Valve Academic Research Consortium
